# The impact of cinacalcet in the mineral metabolism markers of patients on dialysis with severe secondary hyperparathyroidism

**DOI:** 10.1590/2175-8239-JBN-2018-0219

**Published:** 2019-07-18

**Authors:** Sérgio Gardano Elias Bucharles, Fellype Carvalho Barreto, Miguel Carlos Riella

**Affiliations:** 1 Universidade Federal do Paraná Hospital de Clínicas CuritibaPR Brasil Universidade Federal do Paraná, Hospital de Clínicas, Curitiba, PR, Brasil.; 2 Fundação Pró-Renal CuritibaPR Brasil Fundação Pró-Renal, Curitiba, PR, Brasil.

**Keywords:** Calcimimetic Agents, Hyperparathyroidism, Secondary, Kidney Failure, Chronic, Renal Dialysis, Peritoneal Dialysis

## Abstract

**Introduction:**

Treating secondary hyperparathyroidism (SHPT), a common condition associated with death in patients with chronic kidney disease, is a challenge for nephrologists. Calcimimetics have allowed the introduction of drug therapies no longer based on phosphate binders and active vitamin D. This study aimed to assess the safety and effectiveness of cinacalcet in managing chronic dialysis patients with severe SHPT.

**Methods:**

This retrospective study included 26 patients [age: 52 ± 12 years; 55% females; time on dialysis: 54 (4-236) months] on hemodialysis (N = 18) or peritoneal dialysis (N = 8) with severe SHPT (intact parathyroid hormone (iPTH) level > 600 pg/mL) and hyperphosphatemia and/or persistent hypercalcemia treated with cinacalcet. The patients were followed for 12 months. Their serum calcium (Ca), phosphorus (P), alkaline phosphatase (ALP), and iPTH levels were measured at baseline and on days 30, 60, 90, 180, and 365.

**Results:**

Patients with hyperphosphatemia (57.7%), hypercalcemia (23%), or both (19.3%) with iPTH > 600 pg/mL were prescribed cinacalcet. At the end of the study, decreases were observed in iPTH (1348 ± 422 vs. 440 ± 210 pg/mL; *p* < 0.001), Ca (9.5 ± 1.0 vs. 9.1 ± 0.6 mg/dl; *p* = 0.004), P (6.0 ± 1.3 vs. 4.9 ± 1.1 mg/dl; *p* < 0.001), and ALP (202 ± 135 vs. 155 ± 109 IU/L; *p* = 0.006) levels. Adverse events included hypocalcemia (26%) and digestive problems (23%). At the end of the study, 73% of the patients were on active vitamin D and cinacalcet. Three (11.5%) patients on peritoneal dialysis did not respond to therapy with cinacalcet, and their iPTH levels were never below 800 pg/mL.

**Conclusion:**

Cinacalcet combined with traditional therapy proved safe and effective and helped manage the mineral metabolism of patients with severe SHPT.

## Introduction

Chronic kidney disease-mineral and bone disorder (CKD-MBD) is one of the main metabolic disorders associated with chronic kidney disease (CKD) on account of its links with increased risk of cardiovascular events, fractures, and death.^1^^,^^2^ In this context, secondary hyperparathyroidism (SHPT) emerges as one of the main complications endured particularly by patients with advanced-stage CKD on renal replacement therapy. 3

The pathogenesis of SHPT in individuals with CKD is complex and involves different factors such as hypocalcemia, hyperphosphatemia, and nutritional vitamin D and calcitriol deficiency. Other factors have been recently assigned roles in the pathophysiology of the condition, including increased serum levels of fibroblast growth factor 23 (FGF-23) and sclerostin, decreased expression of vitamin D receptors (VDR), FGF-23 receptors (FGFR), Klotho, and calcium-sensing receptor (CaSR), all in parathyroid tissue. Altogether, these factors contribute to increases in the synthesis and secretion of parathyroid hormone (PTH) by parathyroid chief cells. 4

Traditionally, SHPT therapy revolved around maintaining normal calcium and phosphorus levels and managing PTH levels with the help of calcium-based or non-calcium-based phosphate binders, nutritional vitamin D supplementation, and active vitamin D (VDR activation).^2^^,^^4^ However, these measures have limited effect for a significant proportion of patients and have been associated with increased risk of hypercalcemia and hyperphosphatemia, which by their turn have been linked with increased mortality and cardiovascular disorders such as endothelial dysfunction and vascular calcification. 4 Additionally, parathyroidectomy cannot be universally performed on patients with severe disease or be categorized as a risk-free, perfect solution for all cases. 5 The census survey published by the Brazilian Society of Nephrology in 2011 found that approximately 11% of the patients with CKD had severe SHPT (PTH > 1000 pg/mL), indicating that a significant portion of the patients prescribed traditional drug therapy might require parathyroidectomy for being clinically untreatable. 6

The introduction of calcimimetics (cinacalcet) in nephrology care was a major development in the treatment of SHPT. 7 However, there is no definitive evidence indicating that a specific drug class or the combination of calcimimetics and VDR activators might be preferred to manage the PTH levels of patients on renal replacement therapy, be it hemodialysis (HD) or peritoneal dialysis (PD). 8 The more recent guidelines for the treatment of CKD-MBD suggest that (i) the initial choice of drug therapy for SHPT should be based on the potential interactions with other medications used concomitantly and the biochemical profile of the patient (calcium and phosphorus levels) at the time of clinical examination;^9^^,^^10^ and that (ii) treatment may be based on vitamin D analogues, calcimimetics, or a combination of both.

Cinacalcet acts as an allosteric activator of CaSR, thereby increasing the sensitivity of CaSR to extracellular calcium and promoting decreased PTH secretion without increasing serum calcium and phosphorus levels.^11^^,^^12^ Therefore, it can be prescribed even to individuals with hypercalcemia and/or hyperphosphatemia, a situation in which active vitamin D and its analogues are contraindicated.

This study aimed to retrospectively analyze the effectiveness and safety of cinacalcet in patients with CKD on HD or PD with severe SHPT refractory to conventional therapy, phosphate binders, and active vitamin D.

## Methods

This retrospective study was carried out in a CKD-MBD outpatient clinic in Curitiba, in the southern Brazilian state of Paraná, to which patients on chronic dialysis with severe SHPT (PTH persistently > 800 pg/mL) refractory to traditional therapy are referred for treatment with cinacalcet. Hyperphosphatemia was defined as serum phosphorus > 4.7 mg/dL and hypercalcemia as serum calcium > 10.3 mg/dL (upper limits of the testing methods). The measurements of calcium, phosphorus, PTH (radioimmunoassay, reference values 12-65 pg/mL), alkaline phosphatase, and 25-hydroxyvitamin D (DiaSorin LIAISON 25OH vitamin D assay, DiaSorin Inc. Stillwater, Minnesota, normal reference values 30-60 ng/mL) levels were performed using well-known methods previously described in the literature.^2^^,^^13^

The study included all clinically stable patients aged 18+ years on hemodialysis (HD) or peritoneal dialysis (PD) for at least three months referred to the CKD-MBD outpatient clinic at Fundação Pró-Renal (Curitiba, PR, Brazil) needing treatment with calcimimetics or parathyroidectomy for severe SHPT associated with persistent hyperphosphatemia and/or hypercalcemia between January of 2015 and December of 2016.

Patients referred to renal transplantation, parathyroidectomy, and the individuals who died during the study were excluded (patients followed for less than 12 months). Demographic, clinical, and workup information were collected at baseline and traditional mineral metabolism parameters (calcium, phosphorus, PTH) were analyzed on days 30, 60, 90, 180, and 365 of therapy with cinacalcet. Additionally, cinacalcet doses and parameters associated with safety (digestive problems, episodes of hypocalcemia), effectiveness, dialysate calcium concentration, and information concerning the concomitant use of phosphate binders and vitamin D analogues were recorded. Regardless of PTH level, all patients started treatment with 30 mg/day of cinacalcet. The patients included in the study underwent semi-quantitative assessment of vascular calcification by plain x-ray at baseline (Adragao score). 14

### Statistical analysis

Quantitative variables were described in terms of mean values, standard error, medians, and amplitude. Categorical variables were described in terms of frequencies and proportions. Comparisons between quantitative variables at different times were performed based on analysis of variance (ANOVA) with repeat measures or with the Friedman test. Variable distribution normality was tested with the Kolmogorov-Smirnov test. The binomial test was used to compare nominal dichotomous variables at two different times. Comparisons between quantitative variables from two groups were performed with Student’s t-test. Categorical variables were compared based on Fisher’s exact test. Statistical significance was attributed to *p*-values < 0.05. The data were treated on software package IBM SPSS Statistics v.20.0. Armonk, NY; IBM Incorporation.

## Results

Table 1 shows a selection of demographic data and the clinical and biochemical parameters at baseline of the patients followed for 12 months. Forty-two patients were included at first, 30 on HD and 12 on PD. Six died during follow-up (all for cardiovascular events), six underwent renal transplantation, and four were submitted to parathyroidectomy, which means 16 were excluded. The mean age of the group was 52 ± 12 years, 55% were females, the median time on renal replacement therapy was 54 months, and 30% had diabetes. Mean baseline PTH level was 1348 ± 422 pg/mL; mean serum calcium was 9.5 ± 1.0 mg/dL; and mean serum phosphorus was 6.0 ± 1.3 mg/dL. Only two of 26 patients had 25(OH)D_3_ levels > 30 ng/mL at baseline. Cinacalcet was prescribed based on the following findings: persistent hyperphosphatemia (15 patients); recurrent hypercalcemia (6 patients); and persistent hyperphosphatemia + recurrent hypercalcemia (5 patients) accompanied by PTH levels > 600 pg/mL. The patients had been off active vitamin D for at least 30 days at baseline. In regards to phosphate binders, 12 patients were on sevelamer hydrochloride; seven were on calcium carbonate; and seven were on a combination of the two phosphate binders.

**Table 1:** Baseline demographic, clinical, and workup parameters of patients treated with cinacalcet for 12 months

Variables	Results N = 26
Age (years)	52 ± 12
Female sex (%)	55%
Time on RRT (months)	54 (4-236)
Patients with diabetes (%)	30%
Dialysis mode (number - HD/PD)	18 HD / 8 PD
Race (Caucasians – %)	92%
Adragao Score (median / minimum-maximum)	1 (0-3)
Calcium (mg/dL)	9.5 ± 1.0
Phosphate (mg/dL)	6.0 ± 1.3
Parathyroid hormone (pg/mL)	1348 ± 422
Alkaline phosphatase (UI/L)	202.7 ± 135.9
25-hydroxyvitamin D (ng/mL)	18.2 ± 6.1

RRT:renal replacement therapy; HD:hemodialysis; PD:peritoneal dialysis

Patients on HD were treated with a dialysate calcium concentration of 2.5 mEq/L. Five patients on PD were treated with a dialysate calcium concentration of 2.5 mEq/L and three with a dialysate calcium concentration of 3.5 mEq/L. According to the Adragao Score, five patients had no vascular calcification (score = 0), eleven had mild calcification (score = 1), eight had moderate calcification (score = 2), and two had severe calcification (score = 3). The initial mean dose of cinacalcet prescribed was 30 mg/day. Prescribed doses were gradually increased to 41 ± 15 mg on day 60 of follow-up (15-60 mg), 48 ± 23 mg on day 90 of follow-up (0-90 mg), 51 ± 21 mg on day 180 of follow-up (30-90 mg), and 51 ± 28 mg (0-120 mg) on day 365 of follow-up (*p* = 0.03).

**Figure 1:**
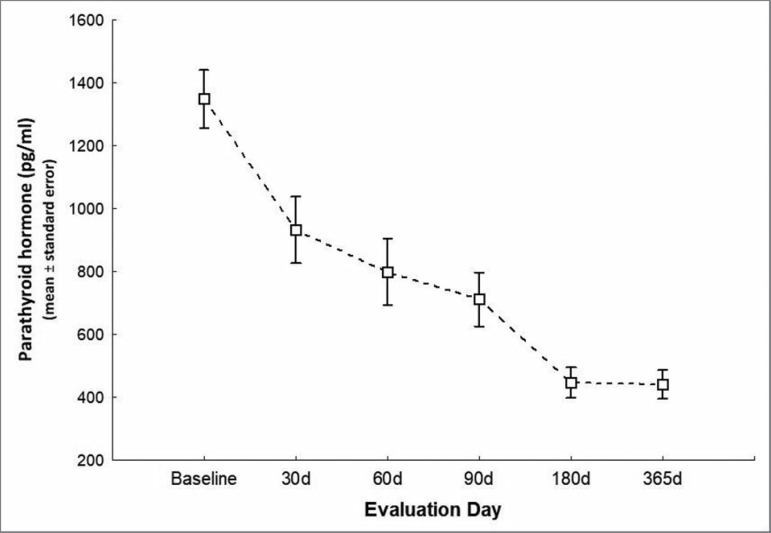
Parathyroid hormone levels during the study.

Table 2 presents the main data concerning patient follow-up and treatment. None of the patients had PTH levels within the preferred target range (150-600 pg/mL) at baseline. On days 90, 180, and 365 of follow-up, 11/25 (44%), 17/26 (65%), and 21/26 (80%) patients, respectively, were within the target range for PTH (*p* = 0.004, levels on days 180 and 365 vs. baseline levels). Mean PTH levels decreased significantly during follow-up to reach 711 ± 394 pg/mL on day 90, 446 ± 221 pg/mL on day 180, and 440 ± 210 pg/mL on day 365 (*p* < 0.001 - Figure 1).

**Table 2:** Workup parameters and clinical events - baseline vs. days 90, 180, and 365 of follow-up

Variable	Baseline	Day 90	Day 180	Day 365
PTH (150-600 pg/mL)	0/26	11/25	17/26	21/26
P (2.6-4.7 mg/dL)	5/26	6/25	6/26	14/26
Ca (8.4-10.3 mg/dL)	16/26	21/25	20/26	23/26
Hypocalcemia	0/26	4/25	7/26	2/26
Active vitamin D	0/26	16/25	17/26	19/26
Nausea and vomiting	0/26	4/25	5/26	6/26

**Figure 2:**
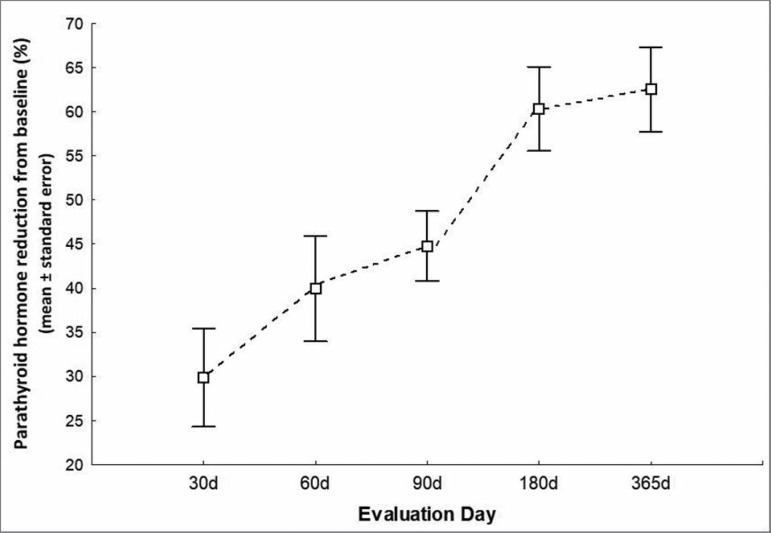
Percent decreases of PTH levels during the study.

**Figure 3:**
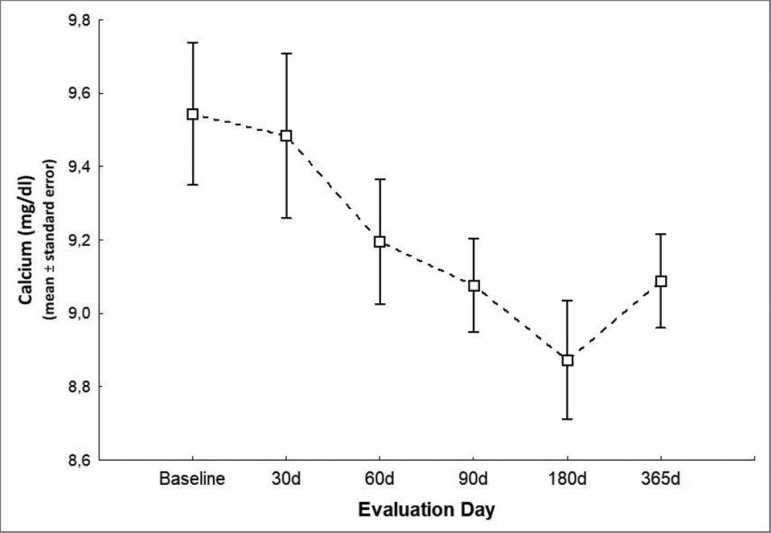
Serum calcium levels during the study.

**Figure 4:**
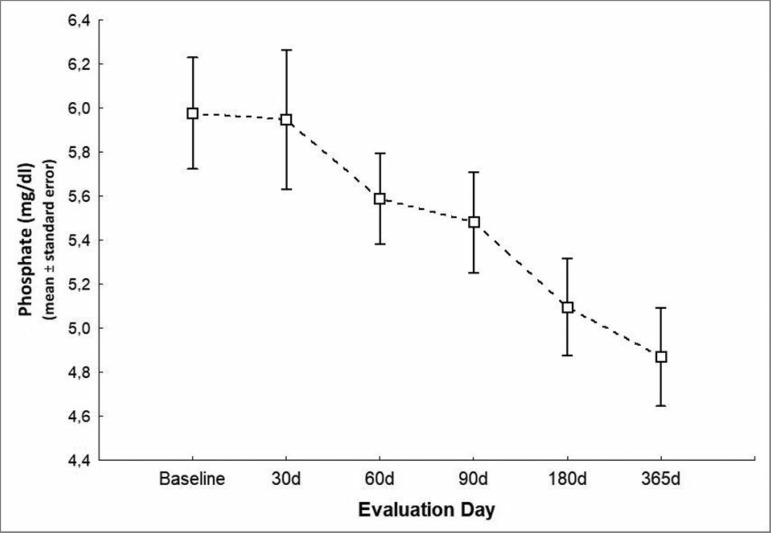
Serum phosphate levels during the study.

**Figure 5:**
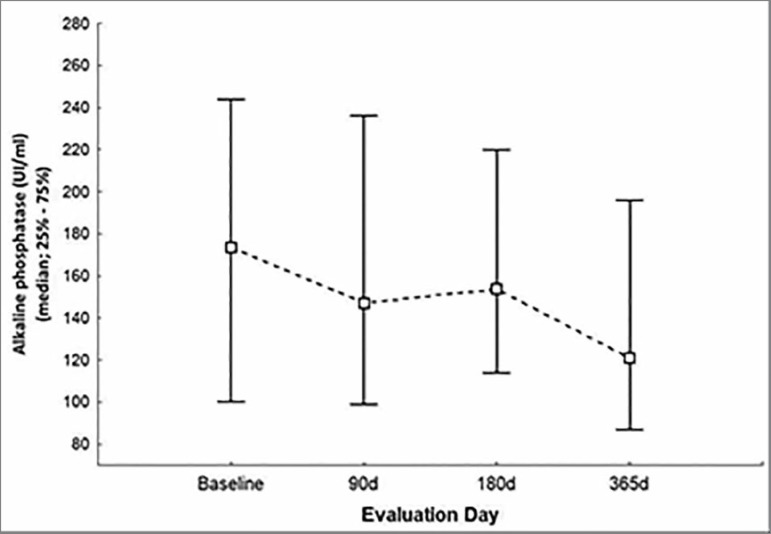
Alkaline phosphatase levels (IU/mL) during the study.

At the end of follow-up, the three patients on peritoneal dialysis still had PTH levels > 800 pg/mL and were on the parathyroidectomy waiting list. The mean PTH level percent decrease relative to baseline was 44.8 ± 19.8% on day 90, 60.3 ± 24.3% on day 180, and 62.5 ± 24.2% at the end of 12 months (Figure 2). Similarly, mean calcium levels decreased significantly relative to baseline to 9.1 ± 0.6 mg/dL on day 90, 8.9 ± 0.8 mg/dL on day 180, and 9.1 ± 0.6 mg/dL on day 365 of follow-up (*p* = 0.004 - Figure 3). Phosphorus levels also decreased relative to baseline (*p* < 0.001 - Figure 4) to 5.5 ± 1.1 mg/dL on day 90, 5.1 ± 1.1 mg/dL on day 180, and 4.9 ± 1.1 mg/dL on day 365. However, 12 of 26 patients still had P levels > 4.7 mg/dL at the end of the study. Alkaline phosphatase also decreased significantly after 12 months of follow-up (202 ± 135 vs. 155 ± 109 IU/L; *p* = 0.006 - Figure 5).

Hypocalcemia (serum calcium < 8.4 mg/dL) was seen in 4/25 patients (16%) on day 90, in 7/26 patients (26%) on day 180, and in 2/26 patients (7%) on day 365 of the study. One patient had severe hypocalcemia (serum calcium < 7.5 mg/dL) and had medication temporarily suspended. The proportion of patients with hypocalcemia did not differ significantly throughout the study on days 90, 180, and 365 (*p* = 0.50). The most common measures to correct hypocalcemia were calcium supplementation with calcium carbonate (4/26 patients) and active vitamin D (14/26 patients). No significant difference was seen in the proportion of patients given active vitamin D throughout the study (*p* = 0.70). At the end of follow-up, 23% of the patients reported vomiting and nausea, particularly when higher doses of cinacalcet were administered (6/26 patients at the end of the study). They were managed with increased doses of proton pump inhibitors and antiemetic medication. The patients were advised to take the medication at the time of the main meal of the day. Variables age, sex, time on renal replacement therapy, dialysis mode, and baseline PTH levels were not independently associated with not achieving PTH levels of 150-600 pg/mL at the end of the study (*p* > 0.05). Most of the patients who stayed until the end of the study were on active vitamin D (19/26 patients).

## Discussion

The main findings of our study supported the safety and efficacy data published previously on the use of cinacalcet in patients with CKD and SHPT.^12^^,^^15^ Cinacalcet effectively controls and sustains calcium, phosphorus, and PTH levels in a significant portion of patients^7^^,^^16^ and offers an acceptable rate of complications including hypocalcemia and digestive problems. 17 Additionally, trials have reported concurrent decreases in serum FGF-23 levels^18^^,^^19^ and lower bone formation rates based on biopsy and bone histomorphometry findings - typically seen in more severe cases of SHPT 20, which combined are therapeutic targets associated with increased mortality in patients with advanced CKD. 1

Hypovitaminosis D - a frequent finding in patients with advanced CKD 13 - was seen in 92% of the individuals included in our study. Although hypovitaminosis D therapy with cholecalciferol has been recommended in the international CKD-MBD guidelines to patients with disease of any stage, in general lines this intervention does not improve the SHPT status of patients on dialysis. 21 However, treatment for hypovitaminosis D is nonetheless offered to patients on dialysis followed on an outpatient basis in our institution in agreement with published protocols and studies, not only to improve bone metabolism, but also for the potential pleiotropic effects of vitamin D. 22

Vascular calcification is a frequent complication among patients on dialysis characterized by complex pathophysiology and close ties with increased mortality. 23 Eighty percent of our patients had vascular calcifications, which ranged from mild to moderate in most cases. Patients with SHPT must be examined for vascular calcification, since it may direct the course of therapy toward the combined use of calcimimetics and low-dose vitamin D analogues to decelerate the progression of the disease. 24

In our study, ongoing use of gradually higher doses of cinacalcet (51.9 ± 28 mg at 12 months of follow-up) allowed most of the treated patients to achieve PTH levels of 150-600 pg/mL, the target range recommended in the more recent international guidelines for CKD-MBD 9 regardless of baseline PTH levels and other variables such as age, sex, and time on renal replacement therapy as reported in previous studies.^12^^,^^15^^,^^24^^,^^25^ Mean PTH levels decreased significantly during the study to 711 ± 394 pg/mL on day 90, 446 ± 221 pg/mL on day 180, and 440 ± 210 pg/mL on day 365 of the study (*p* < 0.001 - Figure 1). The mean PTH level decrease at the end of the study compared with baseline was 62.5% (Figure 2).

A number of published studies have indicated the superiority of combined therapy with calcimimetics and vitamin D analogues vis-à-vis traditional schemes (phosphate binders and active vitamin D) at achieving improved control over the biochemical parameters of individuals with CKD-MBD. 17 However, improved control does not necessarily translate into lower rates of parathyroidectomy to treat SHPT for a significant proportion of patients^6^^,^^26^ who probably were not offered adequate treatment in the earlier stages of the disease.

Cinacalcet is an allosteric activator of CaSR that decreases the secretion and synthesis of PTH in the parathyroid glands, often decreasing serum calcium and phosphorus levels.^15^^,^^27^ In clinical terms, the changes observed in workup parameters are similar to the alterations seen in hungry bone syndrome, a condition often observed in dialysis patients not submitted to parathyroidectomy for severe SHPT, 28 characterized by avid bone resorption of calcium and phosphorus favoring mineralization. However, while parathyroidectomy patients develop significant abrupt hypocalcemia that calls for immediate treatment, hypocalcemia associated with the use of cinacalcet is less pronounced and progresses more slowly. 29 In fact, different studies have reported that approximately 50% of dialysis patients on calcimimetics have at least one episode of hypocalcemia.^30^^,^^31^ In our study, 26% of the patients had serum calcium levels < 8.4 mg/dL at some point. A possible explanation for the difference is that throughout the EVOLVE trial only 17% of the patients with hypocalcemia were given active vitamin D to manage the complication, 32 while the patients in our study were prescribed active vitamin D whenever their serum calcium and phosphorus levels allowed it.

All patients with PTH levels > 800 pg/mL at the end of follow-up were on PD. These patients, without exception, were on the parathyroidectomy waiting list and had not undergone the procedure yet on account of the long wait for surgery, a common scene in CKD-MBD treatment centers. 6 Another study reporting on PD patients given cinacalcet saw significant decreases in PTH levels early on, which however were not sustained six months into follow-up. 33 According to the authors, the high prevalence of digestive problems in the study (77%) might have contributed to the failure of therapy in most cases. Other studies enrolling individuals on PD did not report as many instances of treatment failure or complication. 34

Patients with persistently high PTH levels (PTH > 800 pg/mL for longer than six months) despite adequate drug therapy (maximum tolerated dose of vitamin D analogues and calcimimetics combined) often accompanied by hyperphosphatemia and/or hypercalcemia meet the criteria for parathyroidectomy. 16 Presence or high risk of calciphylaxis, hyporesponsiveness to anemia therapy with erythropoietin (patients with persistently high PTH levels), and parathyroid glands with volumes > 500 mm 3 or greater diameters > 1 cm assessed through imaging are strongly suggestive of nodular transformation and have been associated with low probability of response to drug therapy. 35 In our CKD-MBD service, ultrasound examination of the anterior cervical region is not routinely performed to estimate parathyroid volume or to predict responsiveness to calcimimetics. Patients are referred to parathyroidectomy based on traditional clinical and workup parameters described in the literature. Additionally, the patients included in the study who underwent parathyroidectomy before the 12-month course of calcimimetics were excluded from the final analysis (as per the exclusion criteria of the study). We recognize that although access to the best clinical therapy possible was ensured, a significant proportion of the patients with severe SHPT - approximately 15% after ten years of dialysis and 38% after 20 years of dialysis 36 - will not respond to therapy and will require parathyroidectomy at some point in the future to resolve the condition. 36

Digestive problems (nausea and vomiting) affected 23% of the patients across dialysis modes. The patients were managed with proton pump inhibitors and antiemetic medication, and were advised to take the medication at the time of the main meal of the day. 37 Adverse gastrointestinal events are the most common secondary effect associated with the use of calcimimetics. Most patients suffer from transient mild to moderate nausea and vomiting - and diarrhea, albeit less frequently - at times mistaken for the effects arising from phosphate binders. 37

Although phosphorus levels generally improved, at the end of the study 12/26 patients (46%) still had serum phosphorus levels > 4.7 mg/dL (hyperphosphatemia), as also found in other studies. 15 Hyperphosphatemia is a common finding among patients on chronic dialysis associated to increased morbidity and mortality. 1 On account of its multifactorial nature, 38 the condition requires a complex treatment protocol that includes low dietary phosphorus intake, appropriate dialysis, and the prescription of phosphate binders. The onset of hyperphosphatemia is only partially linked to increased bone resorption promoted by SHPT. As seen in our study, satisfactory disease resolution cannot be expected to arise from improved management of PTH levels.

Alkaline phosphatase is a biochemical marker of bone formation correlated with bone remodeling histomorphometry parameters directly associated with increased morbimortality of patients on chronic dialysis.^39^^,^^40^ Although bone-specific alkaline phosphatase is a marker often used in trials looking into cinacalcet, we were unable to measure its levels on account of financial limitations. Instead, total alkaline phosphatase was used as a marker of bone formation. During the study, significant decreases were seen in alkaline phosphatase levels relative to baseline (*p* = 0.006 - Figure 5), as also observed in PTH levels. As mentioned above, we were unable to use bone-specific alkaline phosphatase as a marker of bone formation. However, patients with hepatitis B and C and other clinically active liver diseases were excluded, thus increasing the reliability of total alkaline phosphatase as a bone formation marker.

The main limitations of this study include the fact that it is a retrospective analysis performed in one CKD-MBD center in the southern Brazilian city of Curitiba, the absence of a control group, and the short period for which the patients were followed (12 months). Nonetheless, it was one of the first studies to look into the effects of cinacalcet in Brazilian patients with advanced CKD and severe SHPT and to corroborate claims of efficacy and safety of the medication in our region.

## Conclusions

In our study, the administration of cinacalcet to dialysis patients with CKD and severe SHPT was safe and effective, and led to gradual sustained significant decreases in the mean levels of PTH, calcium, and ALP throughout the 12 months for which the patients were followed. Although mean P levels decreased significantly, at the end of the study 46% of the patients on dialysis still had hyperphosphatemia. Treated patients experienced known secondary effects of the medication to an extent similar to individuals included in previous studies.
